# A retrospective study in tumour characteristics and clinical outcomes of overweight and obese women with breast cancer

**DOI:** 10.1007/s10549-022-06836-5

**Published:** 2022-12-28

**Authors:** Carla Luís, João Dias, João Firmino-Machado, Rute Fernandes, Deolinda Pereira, Pilar Baylina, Rúben Fernandes, Raquel Soares

**Affiliations:** 1 Biochemistry Unit, Department of Biomedicine, Faculty of Medicine, University of Porto (FMUP), Al Prof Hernâni Monteiro, 4200-319 Porto, Portugal; 2 i3S - Instituto de Inovação e Investigação em Saúde, University of Porto, Porto, Portugal; 3 Laboratory of Medical and Industrial Biotechnology, Porto Research, Technology, and Innovation Centre (LABMI‐PORTIC), Porto Polytechnic, Porto, Portugal; 4 Portuguese Oncology Institute of Porto (IPO-Porto), Porto, Portugal; 5 EPIUnit–Instituto de Saúde Pública, University of Porto, Porto, Portugal; 6 Departamento de Ciências Médicas, University of Aveiro, Aveiro, Portugal; 7 Centro Académico Clínico Egas Moniz, Aveiro, Portugal; 8 School of Health, Polytechnic of Porto (ESS/P.PORTO), Porto, Portugal; 9 Faculty of Health Sciences, University Fernando Pessoa, Fernando Pessoa Hospital-School (FCS/HEFP/UFP), Porto, Portugal

**Keywords:** Retrospective study, Breast cancer, Obesity, BMI, Clinical outcomes, Tumour characteristics

## Abstract

**Introduction:**

Obesity and breast cancer are two major pathologies closely associated with increasing incidence and mortality rates, especially amongst women. The association between both diseases have been thoroughly discussed but much is still to uncover.

**Aim:**

The aim of this study is to analyse tumour characteristics and clinical outcomes of overweight and obese women to disclosure potential associations and better understand the impact of obesity in breast cancer.

**Materials and methods:**

Clinicopathological information of 2246 women were extracted from the institutional database of comprehensive cancer centre in Portugal diagnosed between 2012 and 2016. Women were stratified according to body mass index as normal, overweight, and obese. Patients’ demographic information and tumour features (age, family history, topographic localization, laterality, histological type, and receptor status) were taken as independent variables and overall survival, tumour stage, differentiation grade and bilaterality were considered clinical outcomes.

**Results:**

The main results reveal that overweight and obesity are predominantly associated with worse outcomes in breast cancer patients. Obese patients present larger (*p*-value: 0.002; *OR* 1.422; 95% *CI* 1.134–1.783) and more poorly differentiated tumours (*p*-value: 0.002; *OR* 1.480; 95% *CI* 1.154–1.898) and tend to have lower overall survival although without statistical significance (*p*-value: 0.117; *OR* 1.309; 95% *CI* 0.934–1.833). Overweighted women are more likely to have bilateral breast cancer (*p*-value: 0.017; *OR* 3.076; 95% *CI* 1.225–7.722) than obese women. The results also reveal that overweight women present less distant metastasis (*p*-value: 0.024; *OR* 0.525; 95%*CI* 0.299–0.920). Topographic localization and laterality did not achieve statistical significance.

**Supplementary Information:**

The online version contains supplementary material available at 10.1007/s10549-022-06836-5.

## Introduction

Obesity is closely associated with several types of cancer, including breast cancer [1]. Strong evidence suggests that obesity-related factors such as inflammatory mediators and adipokines modulate metabolic pathways which may promote tumorigenesis and tumour progression [2].

The most common causes of obesity are a poor diet with high energy intake, lack of physical exercise and sedentary lifestyle. Obesity prevalence estimation by the World Obesity Federation calculates that in 2030, 18% of the world population will live with obesity [3]. According to the National Portuguese Health Survey, in 2019, more than half of the adult Portuguese population (53.6%) was overweight or obese [4], and prevalence in obese Portuguese women increased from 48.3 in 1999 to 51.5% in 2019 [4]. The classification of obesity is usually defined by the Body Mass Index (BMI) calculated by the Quetelet index, weight in kilograms (Kg) divided by height in squared metres (m^2^). A BMI between 19 and 25 kg/m^2^ is considered adequate, whereas individuals with a BMI between 25 and 29.9 kg/m^2^ are classified as overweight and higher values are considered obese [5]. Adipose tissue is a metabolically active organ with diverse functions and several cell types. Besides adipocytes, adipose tissue also comprises immune and stromal cells, connective tissue matrix, vessels, and sensory neurons [6]. This cellular network is responsible for the release of several factors such as adipokines, inflammatory mediators, free fatty acids, oestrogens, hypoxia-inducible factors, and growth factors like insulin-like growth factor, that mediate several relevant metabolic pathways in tumorigenesis such as: AMP-activated protein kinase (AMPK), phosphoinositide 3-kinase (PI3K), hypoxia-inducible factor 1α (HIF1α), serine/threonine kinase (AKT), p53 amongst others [2]. Obesity can also contribute to deleterious effects in breast cancer through increased levels of local oestrogen, a product of aromatase activity [7].

Breast cancer is a complex, heterogeneous pathology with an increasing incidence since the 80 s in North America, Oceania, and Europe [8]. Female breast cancer surpassed lung cancer as the most incident cancer worldwide, with an incidence rate in 2020 of 24.5% and being responsible for 15.5% of female cancer deaths [8]. In Portugal, with a female population of roughly 5 million, in 2020, 7000 new cases of breast cancer were diagnosed, and 1800 women died from the disease [8]. Both obesity and breast cancer, share increasing trends. Epidemiological analyses cross talking obesity and breast cancer have been extensively studied and disclosed obesity as a negative prognostic factor associated with poorer outcomes [9–11]. The Women’s Health Initiative Clinical Trial concluded that women with obesity grades I (BMI between 30 and 34.9 kg/m^2^) and II (BMI between 35 and 39. 9 kg/m^2^) had an increased risk of developing breast cancer up to 52 and 86% respectively when compared to normal BMI [12]. A meta-analysis performed by Chan and colleagues reviewed observational studies and randomized control trials associating BMI and breast cancer and concluded that obesity is associated with lower overall and disease-free survival both on pre and postmenopausal women. Comparing women with normal weight and with obesity (calculated before diagnosis) the summary relative risk of mortality was 1.41 for obese women [13].

Though, not all conclusions are consensual, there are still controversial results associating BMI and tumour features, for example, HER2 status. A previous study from Mieghem et al. HER2 was found to be inversely associated with BMI [14] but another study performed by Phipps et al. no correlation was found [15]. Additionally, population characteristics like race [16] and environmental traits like solar exposure [17] can also influence cancer evolution. Herein, we conducted a retrospective cohort study to evaluate the different tumour features and cancer outcomes of women with breast cancer stratified by BMI categories amongst the northern Portuguese population.

## Materials and methods

### Study population and ethical approval

This retrospective cohort study, from the “Deciphering Obesity and Cancer” (DOC) database, included patients diagnosed at Comprehensive Cancer Centre in Portugal (IPO-Porto). Clinicopathological information was extracted from the institutional database in 16, December 2021, with approval of the institutional ethics committee. Women with breast cancer diagnosed between 2012 and 2016 were identified and assessed for inclusion. Male breast cancer as well as underweight women (BMI inferior to 18.5 kg/m^2^) were excluded.

### Methods

BMI was calculated using the Quetelet index, weight and height were measure up to 120 days after diagnosis. BMI was grouped in three categories: normal (18.5 ≤ BMI < 25 kg/m^2^), overweight (25 ≤ BMI < 30 kg/m^2^), and obese (BMI ≥ 30 kg/m^2^) [5]. Independent variables and outcomes of interest were stratified according to BMI categorical distribution.

Age at diagnosis, family history, topographic localization, laterality, histological type, and receptor status were considered as exposure/independent variables. Outcomes of interest was bilaterality, differentiation grade, cancer staging, and overall survival (OS). Independent variable was determined by a patient parameter able to induce an unexpected variation in a measurable outcome [18].

Family history was based on the patient referral to an oncogenetic follow-up in the host institution according to the recommendation statement found in [19].

Topographic localization was divided in inferior outer quadrant (IOQ), inferior inner quadrant (IIQ), superior outer quadrant (SOQ), superior inner quadrant (SIQ), other localizations which included nipple, skin, central quadrant, and axillary extension (Other) and overlapping lesions in different localizations (Multiple).

For histological type, cases were stratified in Invasive Ductal Carcinoma (IDC) which included Invasive Ductal Carcinoma also called no special type (NST) and IDC with other types of carcinomas; Invasive Lobular Carcinoma (ILC) which included Invasive Lobular Carcinoma with other types of carcinomas; and other types of breast cancer (Other).

Each hormonal receptor (estrogen—ER and progesterone—PR) was classified as positive or negative, cut-off of 1% of tumour cells reported as positive. HER2 status was classified according to the American Society of Clinical Oncology/College of American Pathologists (ASCO/CAP) guidelines, a first assessment by immunohistochemistry and in equivocal results, validation by fluorescence in situ hybridization (FISH) [20].

Differentiation grade is classified as grade 1 considered well differentiated, grade 2 for moderately differentiated tumours and grade 3 in poorly differentiated ones. In the present statistical analysis, grade was grouped as High grade (Grades 1 and 2) and Low grade (Grade 3).

Cancer staging (pathologic stage) was assessed accordantly to the American Joint Committee on cancer by tumour node metastasis (TNM) system. Tumour size (T) was divided in ≤ 20 mm and > 20 mm. Lymph node involvement (N) was stratified in the presence (N +) or absence (N0) of nodal infiltration. Likewise for metastasis (M), cases were divided according to the presence or absence of distant metastasis. Overall stage classification was determined by a stage grouping process [21].

Overall survival (OS) was measured in months over 10 years, from the date of diagnosis until the date of death with censoring at the last known follow-up with a cut-off date of 16 December 2021.

### Statistical analysis

Statistical analysis was performed using IBM® SPSS®, version 27 software. Descriptive statistics was used for data description in terms of absolute frequencies and valid percentages. The first approach consisted in a Pearson’s Chi-squared test to analyse the association between BMI and all variables with statistical significance set for *p* < 0.05 obtained by default two-sided qui square test. Age at diagnosis was tested for normality with Shapiro–Wilk test, afterwards a One-way ANOVA and a linear correlation was performed. Independent variables were analysed in binary or multinomial logistic regression in crude and adjusted to age at diagnosis and family history. Topographic localization, histological type and receptor status were analysed with multinomial logistic regression to estimate odd ratio (*OR*) with 95% confidence interval (95% *CI*). For receptor status, each receptor was analysed in separate with a binomial logistic regression model to estimate *OR* with 95% *CI*. Bilaterality, differentiation grade, and each component of the pathological stage was accessed by binomial logistic regression in crude and adjusted to age at diagnosis, family history, histological type, topographic localization, receptor status, and laterality, to estimate *OR* with 95% *CI*. Overall survival analysis was performed by Cox proportional hazard model with a time scale of 10 years (120 months). Estimation was done in crude as well as adjusted to age at diagnosis, family history, histological type, topographic localization, receptor status and laterality, associations were expressed in hazard ratio (*HR*) with 95% *CI*.

## Results

**Fig. 1:**
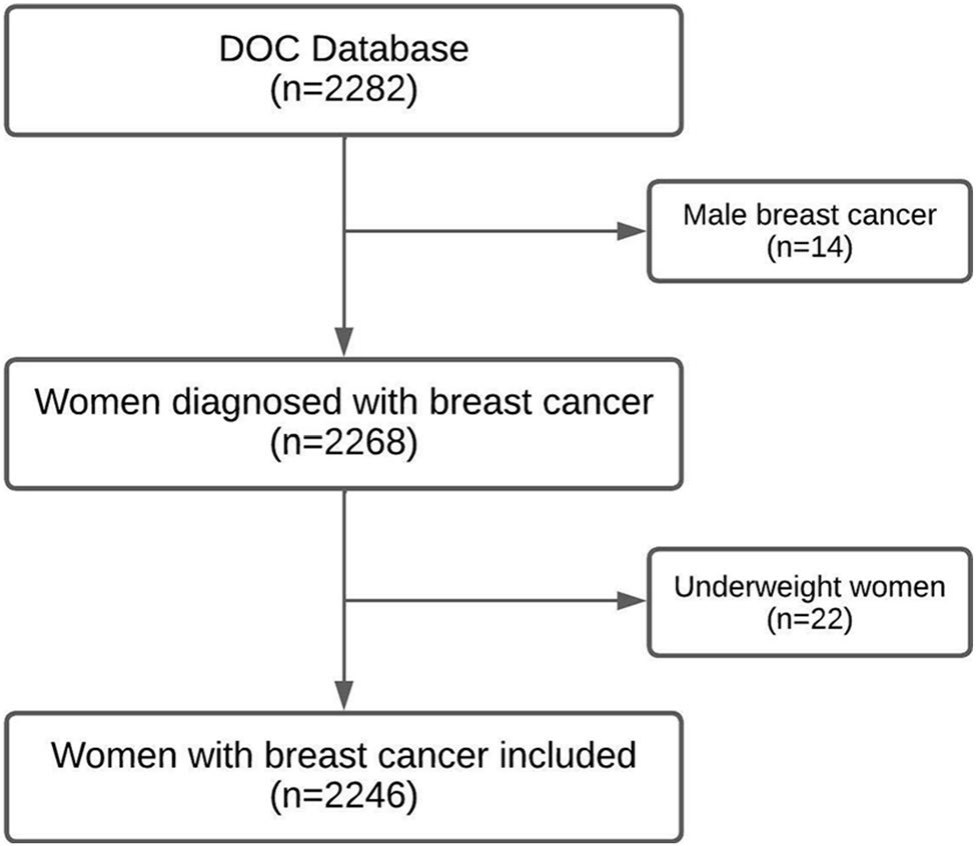
Flowchart of the study inclusion/exclusion criteria Legend: DOC: “Deciphering Obesity and Cancer”

From 2012 to 2016, 2246 women with breast cancer were identified and included in this study, Fig. 1 presents a flowchart with the exclusion criteria applied from the main database.

From the 2246 women, 777 (34.6%) were normal weight, 854 (38.0%) were overweight and 615 (27.4%) were obese. Table 1 presents the demographic and clinicopathological characteristics of women distributed according to BMI categories. There is a significant association between BMI and age at diagnosis, family history, progesterone receptor, receptor status, bilaterality, and tumour stage. It was also found that Invasive ductal carcinoma (IDC) was more frequent in overweight patients. No association was observed in topographic localization, laterality, estrogen receptor, HER2, differentiation grade and overall survival.

**Table 1:** Demographic and clinicopathological characteristics of breast cancer patients stratified by BMI categories

	Normal (BMI 18.5–24.9 kg/m^2^) *n* (%)	Overweight (BMI 25–29.9 kg/m^2^) *n* (%)	Obese (BMI > 30 kg/m^2^) *n* (%)	Total *n* (%)	*p*-value
*n* (%)	777 (34.6%)	854 (38.0%)	615 (27.4%)	2246 (100.0%)	–
Age at diagnosis (Median/range)	50 (24–87)	56 (27–88)	59 (33–86)	55 (24–88)	< 0.001*
Family history					
No	506 (65.1%)	622 (72.8%)	471 (76.6%)	1599 (71.2%)	< 0.001*
Yes	271 (34.9%)	232 (27.2%)	144 (23.4%)	647 (28.8%)	
Topographic Localization					
SIQ	68 (8.8%)	87 (10.2%)	53 (8.6%)	208 (9.3%)	0.666
SOQ	236 (30.4%)	263 (30.8%)	181 (29.4%)	680 (30.3%)	
IIQ	37 (4.8%)	35 (4.1%)	30 (4.9%)	102 (4.5%)	
IOQ	54 (6.9%)	38 (4.4%)	36 (5.9%)	128 (5.7%)	
Other	39 (5.0%)	52 (6.1%)	36 (5.9%)	127 (5.7%)	
Multiple	343 (44.1%)	379 (44.4%)	279 (45.3%)	1001 (44.5%)	
Laterality					
Right	370 (47.6%)	413 (48.5%)	292 (47.5%)	1075 (47.9%)	0.903
Left	407 (52.4%)	438 (51.5%)	323 (52.5%)	1168 (52.0%)	
Missing data	0 (0.0%)	3 (0.1%)	0 (0.0%)	3 (0.1%)	
Histological Type					
IDC	626 (80.6%)	708 (82.9%)	488 (79.3%)	1822 (81.1%)	0.059
ILC	64 (8.2%)	77 (9.0)	49 (8.0%)	190 (8.5%)	
Other	87 (11.2%)	69 (8.1%)	78 (12.7%)	234 (10.4%)	
Estrogen Receptor					
Negative	137 (17.6%)	138 (16.2%)	97 (15.8%)	372 (16.6%)	0.600
Positive	640 (82.4%)	716 (83.8%)	518 (84.2%)	1874 (83.4%)	
Progesterone Receptor					
Negative	253 (32.6%)	243 (28.5%)	158 (25.7%)	654 (29.1%)	0.017*
Positive	524 (67.4%)	611 (71.5%)	457 (74.3%)	1592 (70.9%)	
HER2					
Negative	620 (80.9%)	669 (79.5%)	504 (83.9%)	1793 (81.2%)	0.115
Positive	146 (19.1%)	172 (20.5%)	97 (16.1%)	415 (18.8%)	
Receptor status					
ER + /PR +	443 (57.0%)	510 (59.7%)	394 (64.1%)	1347 (61.0%)	0.032*
ER + /PR-	84 (10.8%)	69 (8.1%)	43 (7.0%)	196 (8.9%)	
HER2 +	146 (18.8%)	172 (20.2%)	97 (15.8%)	415 (18.8%)	
Triple Negative	93 (12.0%)	90 (10.5%)	67 (10.9%)	250 (11.3%)	
Missing data	11 (1.4%)	13 (1.5%)	14 (2.2%)	38 (1.7%)	
Bilaterality					
Unilateral	765 (98.5%)	831 (97.3%)	609 (99.0%)	2205 (98.2%)	0.041*
Bilateral	12 (1.5%)	23 (2.7%)	6 (1.0%)	41 (1.8%)	
Differentiation Grade					
High grade	423 (54.4%)	468 (54.8%)	308 (50.0%)	1199 (53.4%)	0.129
Low grade	348 (44.8%)	376 (44.0%)	303 (49.3%)	1027 (45.7%)	
Missing data	6 (0.8%)	10 (1.2%)	4 (0.7%)	20 (0.9%)	
Tumor Stage					
Stage I	331 (42.6%)	359 (42.1%)	227 (36.9%)	917 (40.9%)	0.029*
Stage II	288 (37.0%)	325 (38.1%)	241 (39.2%)	854 (38.0%)	
Stage III	124 (16.0%)	149 (17.5%)	129 (21.0%)	402 (17.9%)	
Stage IV	34 (4.4%)	20 (2.3%)	18 (2.9%)	72 (3.2%)	
Overall Survival					
No	82 (10.6%)	105 (12.3%)	71 (11.5%)	258 (11.5%)	0.544
Yes	695 (89.4%)	749 (87.7%)	544 (88.5%)	1988 (88.5%)	

*IDC* invasive ductal carcinoma; *ILC* invasive lobular carcinoma; *IOQ* inferior outer quadrant; *IIQ* inferior inner quadrant; *SOQ* superior outer quadrant; *SIQ* superior inner quadrant; *ER* estrogen receptor; *PR* progesterone receptor; *HER2* human epidermal growth factor receptor 2

^*^Statistical significance highlighted in bold (*p* < 0.05);

### Overweight and obese patients are diagnosed later with breast cancer

**Fig. 2:**
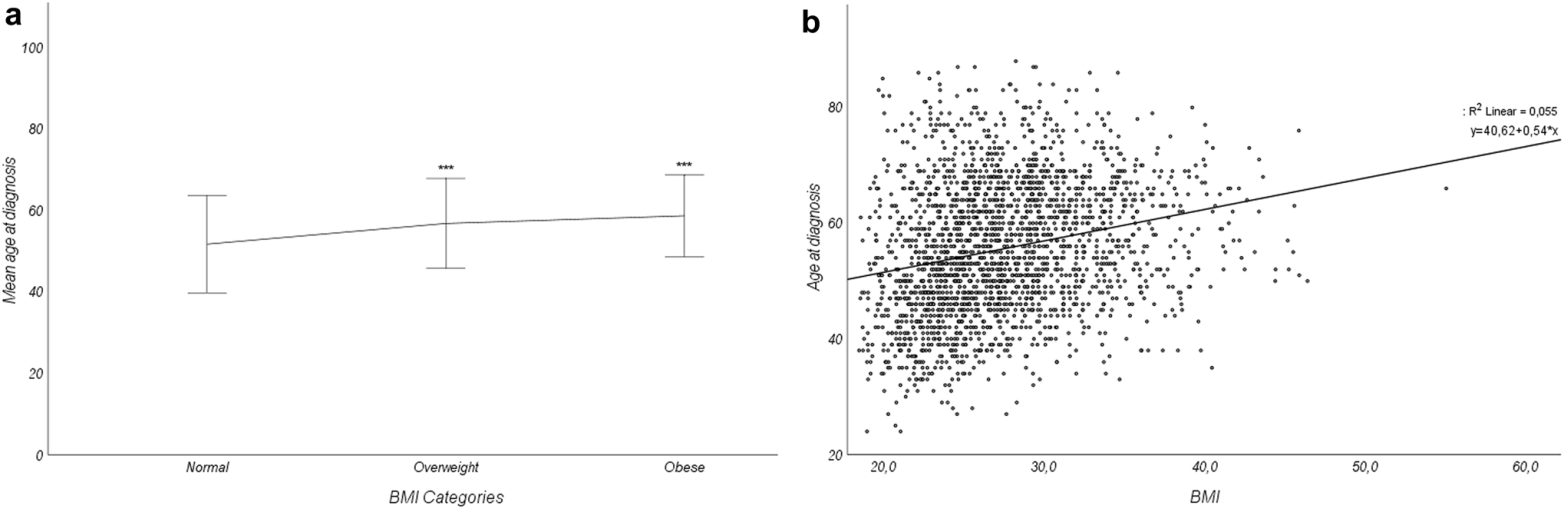
**a** Graphic representation of age at diagnosis by BMI classification in mean ± standard deviation (One-way ANOVA of age at diagnosis and BMI categories, *** *p* < 0.001). **b** Scatter Plot of Age by BMI (*R*^*2*^ = 0.055, *y* = 40.62 + 0.54X)

The mean age of patients at diagnosis was 55.6 ± 11.5 years old with a median of 55.0 (range 24–88). Overweight (age at diagnosis 56.8 ± 11.0) and obese (age at diagnosis: 58.7 ± 10.0) patients were diagnosed later than normoponderal patients (age at diagnosis: 51.7 ± 12.0) (Fig. 2a). Age at diagnosis determined a correlation coefficient of 0.055 (Fig. 2b).

### Family history is less common in obese and overweight patients

**Fig. 3:**
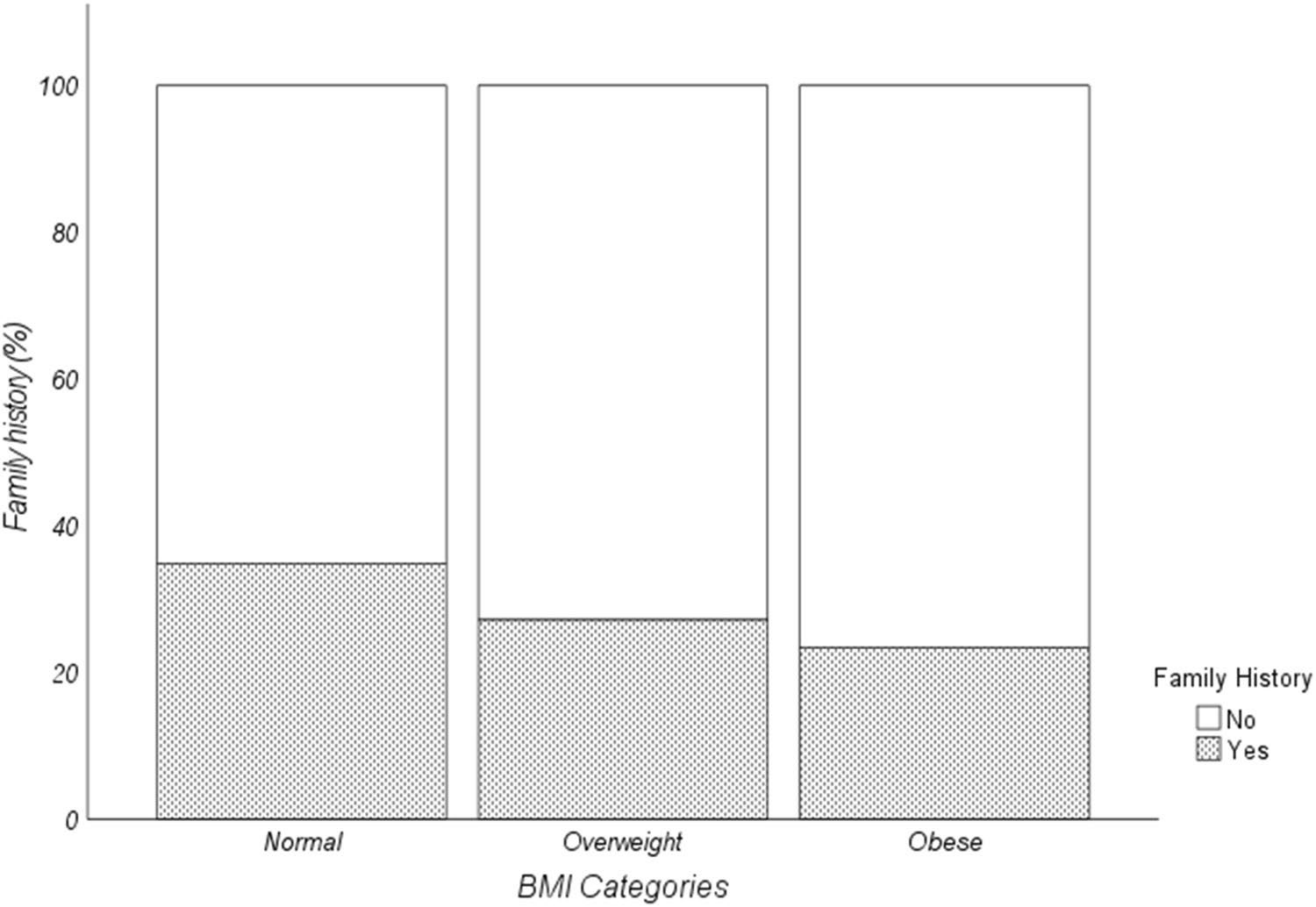
Descriptive analysis of the percentage distribution according to BMI categories of patients with breast cancer and family history (Pearson's Chi-squared test *p*-value: < 0.001)

We observed a statistical association between family history and BMI categories. A descriptive analysis of our cohort observed that absence of family history is more frequent in obese patients (76.6%) when compared to overweight (72.8%) and normal weight (65.1%) subjects (Table 1, *p*-value < 0.001; Fig. 3).

### BMI has no significant association with topographical localization and laterality

**Fig. 4:**
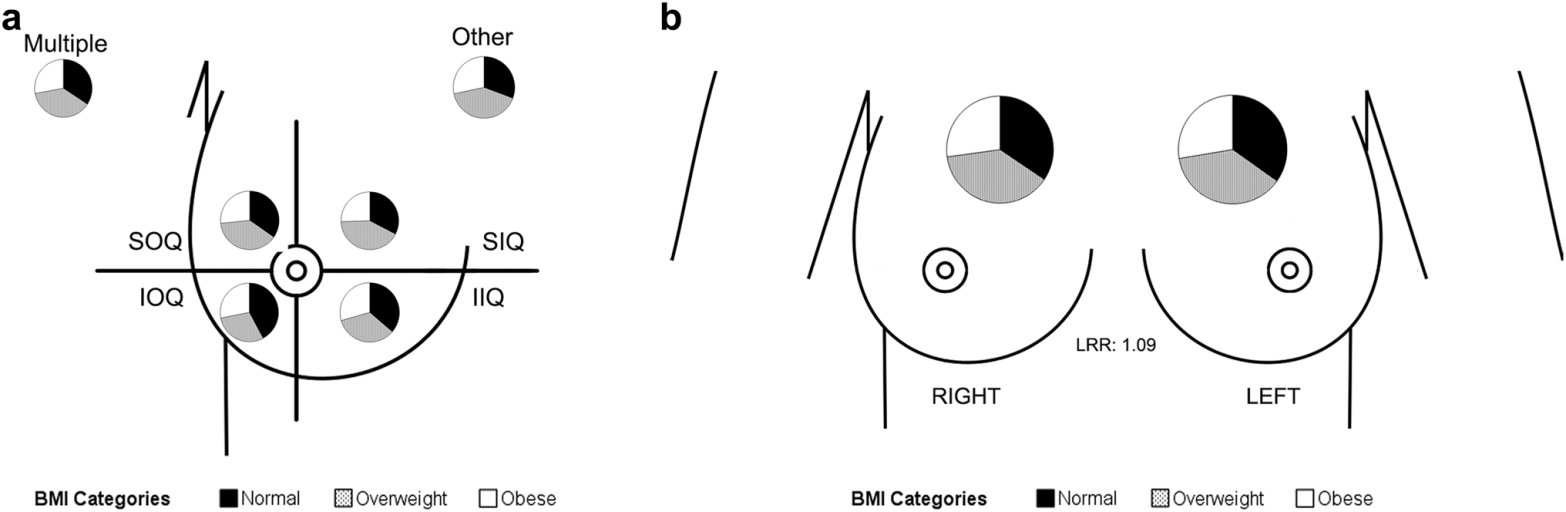
**a** Pie charts of each topographic localization stratified by BMI categories **b** Pie charts of laterality stratified by BMI categories Legend: *IOQ* inferior outer quadrant; *IIQ* inferior inner quadrant; *SOQ* superior outer quadrant; *SIQ* superior inner quadrant; *LRR* left to right ration.

Our results did not reveal a significant association between topographic localization and BMI (Table 1, *p*-value = 0.666) and no alterations in the BMI distribution per quadrant (Fig. 4a). In a multinomial logistic regression adjusted to age at diagnosis and family history, significance remained unachievable (Supplementary Information SI1—Multinomial logistic regression for topographic localization). Association between laterality and BMI distribution did not achieve statistical significance either (Table 1, *p*-value = 0.903) as has the binary logistic regression adjusted to age at diagnosis and family history (Supplementary Information SI2—Binary logistic regression for laterality). The left to right ration (LRR) in our study was 1.09 (Fig. 4b).

### Overweight women are more likely to have ductal invasive carcinoma than other histological type

IDC represents 81.1% of the breast cancer cases and ILC correspond to 8.5% of the participants. No significant association was found across BMI categories (Table 1; *p* value = 0.059). We also analysed qualitatively the distribution of all cases with all other histological types discriminated (Supplementary Information SI3—Description of all histological types stratified by BMI categories). We observed that rare breast cancers with favourable prognosis are more commonly diagnosed in patients with normal weight. For a closest insight of the statistical significance between histological type, we performed a multinomial logistic regression adjusted to age at diagnosis and family history to each reference category (ILC, IDC and Other). We observed that overweight women have increased OR for IDC (*p*-value = 0.030; *OR*: 1.457; 95% *CI* 1.038–2.047; reference: other). Additionally, ILC reference category has no statistical significance with the other groups (Table 2).

**Table 2:** Multinomial logistic regression for Histological Type

		*p*-value	OR	95% *CI*	
				Lower	Upper
Reference category: Other histological type					
IDC	Intercept	< 0.001*	–	–	–
	Normal	–	1	–	–
	**Overweight**	0.030*	**1.457**	**1.038**	**2.047**
	Obese	0.520	0.895	0.638	1.255
ILC	Intercept	0.561	–	–	–
	Normal	–	1	–	–
	Overweight	0.100	1.478	0.927	2.354
	Obese	0.439	0.822	0.501	1.349
Reference category: ILC					
IDC	Intercept	< 0.001*	–	–	–
	Normal	–	1	–	–
	Overweight	0.940	0.926	0.692	1.406
	Obese	0.680	1.088	0.729	1.624
Other	Intercept	0.561	–	–	–
	Normal	–	1	–	–
	Overweight	0.100	0.677	0.425	1.078
	Obese	0.439	1.216	0.741	1.995
Reference category: IDC					
ILC	Intercept	< 0.001*	–	–	–
	Normal	–	1	–	–
	Overweight	0.940	1.014	0.711	1.446
	Obese	0.680	0.919	0.616	1.372
Other	Intercept	< 0.001*	–	–	–
	Normal	–	1	–	–
	**Overweight**	0.030*	**0.686**	**0.489**	**0.963**
	Obese	0.520	1.118	0.797	1.568

Adjusted to age at diagnosis and family history

*CI* confidence interval; *OR* odd ratio

^*^Statistical significance highlighted in bold (*p*-value < 0.05);

### Receptor status: obese women have increased odd ratio to be progesterone receptor positive

Estrogen and progesterone receptors positivity is present in most of our cases (69.9%), HER2 is positive in 18.8% of the cases and triple negative tumours are accountable for 11.3%. Pearson’s *χ*^2^ test reported a significant association between BMI and Receptor Status (Table 1, *p*-value = 0.032).

A binary logistic regression found no significant association in ER and HER2 receptors in the crude and adjusted models, but a significant result was achieved for PR (Table 3). Obese women present an increased odd ratio of 48% (*OR *1.481; 95% *CI* 1.163–1.887) for PR expression in comparison to women with normal weight. Women with overweight have approximately half of this odd ratio, 27%.

**Table 3:** Binary logistic regression for the distinct receptors

	Crude				Adjusted^§^			
	*p*-value	*OR*	95% *CI*		*p*-value	*OR*	95% *CI*	
			Lower	Upper			Lower	Upper
*Estrogen Receptor*								
BMI	0.600	–	–	–	0.672	–	–	–
Normal	–	1	–	–	–	1	–	–
Overweight	0.428	1.111	0.857	1.439	0.469	1.103	0.846	1.437
Obese	0.357	1.143	0.860	1.520	0.420	1.129	0.841	1.514
*Progesterone Receptor*								
BMI	0.017*	–	–	–	0.005*	–	–	–
Normal	–	1	–	–	–	1	–	–
Overweight	0.072	1.214	0.983	1.500	0.031*	**1.268**	**1.022**	**1.574**
Obese	0.005*	**1.397**	**1.104**	**1.767**	0.001*	**1.481**	**1.163**	**1.887**
*HER 2*								
BMI	0.116	–	–	–	0.145	–	–	–
Normal	–	1	–	–	–	1	–	–
Overweight	0.484	1.092	0.854	1.396	0.289	1.146	0.891	1.473
Obese	0.161	0.817	0.616	1.084	0.358	0.873	0.652	1.167

^§^Adjusted to Age at diagnosis and Family history

*OR* odds ratio; *CI* confidence interval

^*^Statistical significance highlighted in bold (*p*-value < 0.05);

### Overweight women are more likely to develop bilateral breast cancer than obese women

In the current study, the BBC incident rate is 1.8%, and with a significant association with BMI (Table 1; *p* = 0.041). The statistical significance persisted with a binary logistic regression analysis (Table 4). Overweight women have an increased non-significant odd ratio of 74.8% (*p*-value: 0.132; *OR* 1.748; 95% *CI* 0.845–3.615) to develop BBC, whereas obese women present a decreased non-significant odd ratio of 43.2% (*p*-value: 0.276; *OR* 0.568; 95% *CI* 0.201–1.570) comparing to normal BMI.

**Table 4:** Logistic binary regression for unilateral and bilateral breast cancer stratified by BMI

	Crude				Adjusted^§^			
	*p*-value	*OR*	95% *CI*		*p*-value	*OR*	95% *CI*	
			Lower	Upper			Lower	Upper
*Reference category: Normal*								
BMI	0.049*	–	–	–	0.037*	–	–	–
Normal	–	1	–	–	–	1	–	–
Overweight	0.114	1.764	0.872	3.570	0.132	1.748	0.845	3.615
Obese	0.355	0.628	0.234	1.683	0.276	0.568	0.206	1.570
*Reference category: Obese*								
BMI	0.049*	–	–	–	0.037*	–	–	–
Obese	–	1	–	–	–	1	–	–
Overweight	0.025*	**2.809**	**1.137**	**6.941**	0.017*	**3.076**	**1.225**	**7.722**
Normal	0.355	1.592	0.594	4.267	0.276	1.759	0.637	4.859

^§^Adjusted to Age at diagnosis, Family history, laterality, Topographic localization, histological type, and Receptor status

*OR* odd ratio; *CI* confidence interval

^*^ Statistical significance highlighted in bold (*p*-value < 0.05);

To better understand the significance of the adjusted model of the binary logistic regression, we performed an identical complementary analysis establishing obese as reference category and found a statistically significant *p*-value of 0.017 (*OR* 3.076; 95% *CI* 1.225–7.722). Comparing to obese women, women with overweight have a threefold increased likelihood to develop BBC.

### Obese women are more susceptible to develop poorly differentiated tumours

Herein, despite no significant association was found between BMI and differentiation grade in the Pearson’s Chi-squared test (Table 1, *p*-value = 0.129), an adjusted binary logistic regression (Table 5) revealed that obesity is significantly associated with tumours with lower differentiation grade (*p*-value = 0.002). Obese women have 48% more likelihood to have poorly differentiated tumours (*OR* 1.480; 95% *CI* 1.154–1.898).

**Table 5:** Logistic binary regression for differentiation grade stratified by BMI

	Crude				Adjusted^§^			
	*p*-value	*OR*	95% *CI*		*p*-value	*OR*	95% *CI*	
			Lower	Upper			Lower	Upper
BMI	0.129	–	–	–	0.002*	–	–	–
Normal	–	1	–	–	–	1	–	–
Overweight	0.813	0.977	0.802	1.188	0.860	1.021	0.814	1.279
Obese	0.100	1.196	0.967	1.479	0.002*	**1.480**	**1.154**	**1.898**

^§^Adjusted to Age at diagnosis, Family history, laterality, Topographic localization, histological type, and Receptor status

*OR* odd ratio; *CI* confidence interval

^*^Statistical significance highlighted in bold (*p*-value < 0.05);

### Obesity is associated with larger primary tumours and less distant metastasis

Our results report that BMI is significant associated with pathological stage (Table 1, *p*-value = 0.029). As illustrated in Table 6, BMI is strongly associated with tumour size, moderately (but not significantly) associated with distant metastasis, and poorly associated with lymph node involvement.

**Table 6:** Descriptive characteristics of tumour stage features by BMI Status with association analysis by Pearson’s Chi-squared test

Characteristics	Normal BMI *n* (%)	Overweight *n* (%)	Obese *n* (%)	Total *n* (%)	*p*-value
Tumour size (T)					
≤ 20 mm	442 (57.2%)	472 (55.5%)	309 (50.2%)	1223 (54.6%)	0.030*
> 20 mm	331 (42.8%)	379 (44.5%)	306 (49.8%)	1016 (45.4%)	
Distant metastasis (M)					
No	743 (95.6%)	833 (97.7%)	597 (97.1%)	2173 (96.8%)	0.060
Yes	34 (4.4%)	20 (2.3%)	18 (2.9%)	72 (3.2%)	
Lymph node involvement (N)					
N0	421 (54.4%)	486 (57.1%)	322 (52.4%)	1229 (54.9%)	0.186
N +	353 (45.6%)	365 (42.9%)	293 (47.6%)	1011 (47.6%)	

*OR* odd ratio; *CI* confidence interval

^*^Statistical significance highlighted in bold (*p*-value < 0.05)

After binary logistic regression for each feature, adjusted models reveal that obese patients have an 42% increased OR (95%: 1.134–1.783) to develop tumours with more than 20 mm. In the unadjusted model, there is a significant reduced OR of approximately 50% (95% *CI* 0.299–0.920) for overweight women to exhibit distant metastasis. Our results did not observe any association with lymph node involvement (Table 7).

**Table 7:** Binary logistic regression for the distinct features of tumour stage

	Crude				Adjusted^§^			
	*p*-value	*OR*	95% *CI*		*p*-value	*OR*	95% *CI*	
			Lower	Upper			Lower	Upper
Tumour size								
Normal	–	1	–	–	–	1	–	–
Overweight	0.486	1.072	0.881	1.305	0.151	1.163	0.946	1.428
Obese	0.010*	**1.322**	**1.069**	**1.636**	0.002*	**1.422**	**1.134**	**1.783**
Distant Metastasis								
BMI	0.065	–	–	–	0.246	–	–	–
Normal	–	1	–	–	–	1	–	–
Overweight	0.024*	**0.525**	**0.299**	**0.920**	0.094	0.611	0.343	1.088
Obese	0.160	0.659	0.368	1.178	0.498	0.811	0.442	1.488
Lymph Node involvement								
BMI	0.186	–	–	–	0.098	–	–	–
Normal	–	1	–	–	–	1	–	–
Overweight	0.271	0.896	0.736	1.090	0.414	0.919	0.750	1.126
Obese	0.450	1.085	0.878	1.342	0.187	1.162	0.930	1.452

^§^Adjusted to Age at diagnosis, Family history, laterality, Topographic localization, histological type, and Receptor status

*OR* odd ratio; *CI* confidence interval

^*^Statistical significance highlighted in bold (*p*-value < 0.05);

### Obese and overweight patients present lower overall survival

**Fig. 5:**
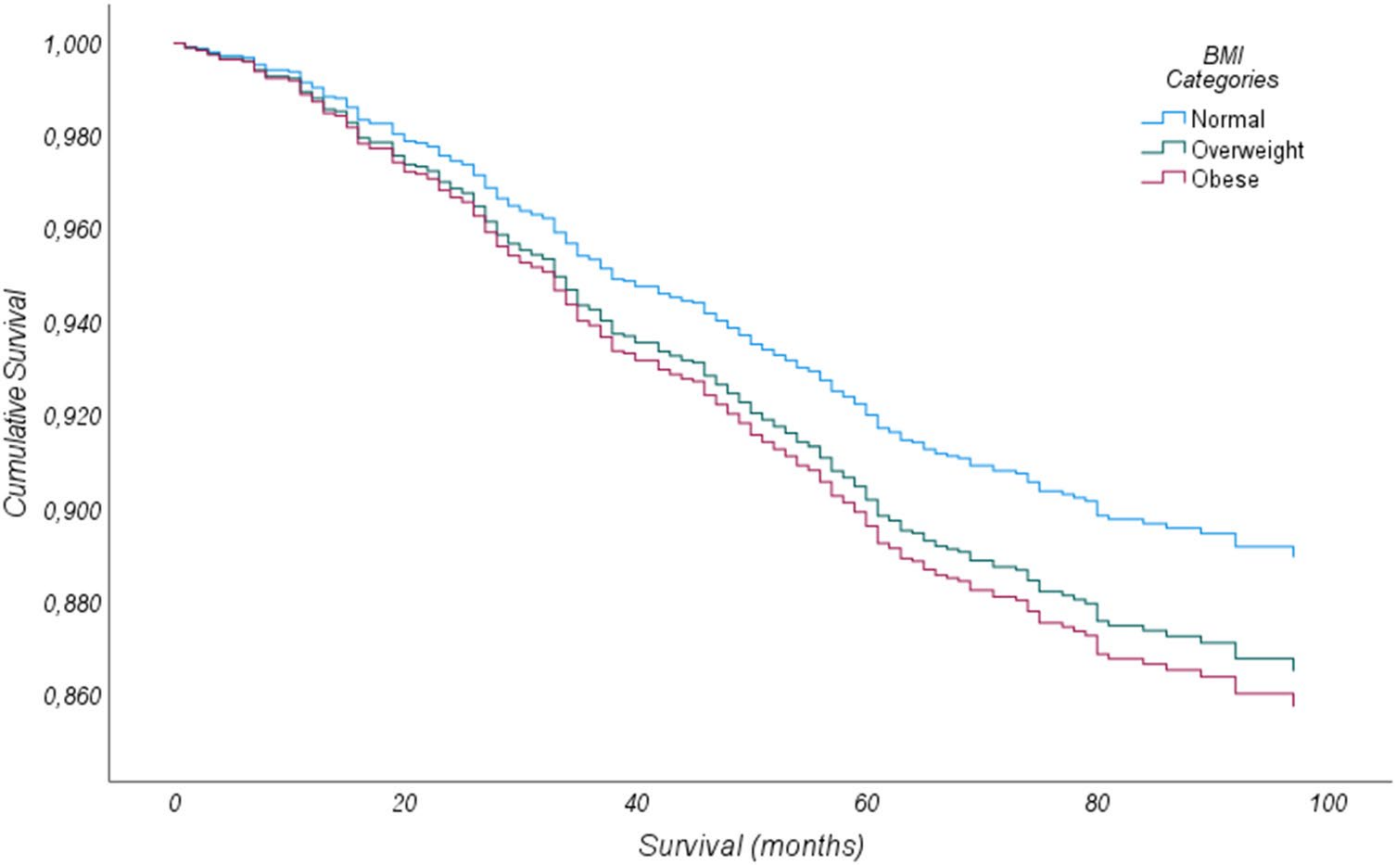
Overall survival by Cox proportional hazard model stratified by BMI categories.

We observed that normal weight women have a better OS compared to women with overweight and obesity. OS of overweight women is close to women with obesity with a slight better outcome (Fig. 5). Although, statistical analysis did not achieve significant difference (Supplementary Information SI4—Cox proportional hazard models for overall survival), the HR for women with overweight and with obesity in the adjusted model was 1.224 (*p*-value = 0.182; 95% *CI* 0.909–1.648) and 1.309 (*p*-value = 95%; *CI* 0.934–1.833) respectively.

## Discussion and conclusion

Breast cancer and obesity are two metabolically associated pathologies with high and increasing prevalence rates, which develop to a complex and challenging clinical concern. Our study intended to uncover associations in obese and overweight breast cancer patients within the northern Portuguese population. Integrating our results within the Portuguese population, we observed that the percentage of obese and overweight patients in the current study exceeds that of the obese/overweight female population (65.4% vs 51.5%). Our results also pointed that overweight and obese patients are diagnosed later that normal weight patients. A trend also observed in other countries, the northern Portuguese population is diagnosed later than countries such as Belgium [22], Arabian countries [23, 24], or Mexico [25], but earlier than in countries like as United States of America (USA) [26]. We postulated that this is attributed to the fact that obesity is often associated with lower social status and lower income, thus, providing patients with less health care opportunities and to a lower adhesion rate to the screening programmes of obese women [27]. Additionally, obesity is accompanied by a low-grade chronic inflammation, increased oxidative stress, hypertension, dyslipidaemia [28], and several other metabolic comorbidities, which may mask cancer symptoms and diagnosis. We also found that the correlation factor of age at diagnosis in our cohort is superior to the correlation found in other studies [22], indicating a stronger relation between age at diagnosis and BMI.

Breast tumorigenesis can be initiated by several factors like genetics [29], reproductive and modifiable factors. Modifiable risk factors include physical inactivity, alcohol consumption and obesity [29]. Our study revealed an association between obesity and sporadic breast cancer—lack of family history. Supporting our results, previous studies found lower family history index in both pre and postmenopausal in obese and overweight women [30].

According to the World Health Organization (WHO) there are 23 histological subtypes of breast cancer [31], the more incident ones are the IDC accountable for 70–80% of all invasive tumours and ILC diagnosed in 5–15% [32], our results are concomitant with the literature, IDC represents 81.1% and ILC corresponding to 8.5%. Our findings uncover that overweight women are more likely to have IDC than other histological type, to the best of our knowledge, no association was previously described regarding histological type and BMI [33].

Each histological type has intrinsic characteristics with different prognostic value, outcomes are modulated by several factors such as histological grade, menopausal status, therapeutical plan, or receptor positivity [32]. The association between breast cancer aetiology and steroid hormone receptors has been thoroughly studied. ER + and PR + are the most common and present a more favourable prognosis and survival advantage [34]. Though, it is suggested that postmenopausal women with overweight and obesity with ER + /PR + tumours exhibit an increased risk of developing breast cancer up to twofold [35]. HER2 + tumours account for ~ 15% of overall breast cancers. Their prognosis has shifted with the introduction of targeted therapies (such as trastuzumab). Triple negative tumours represent 10–20% of breast cancers and are the most aggressive ones, with the worst prognosis amongst breast cancers, these are usually diagnosed at a younger age with a low overall survival [36]. Previous studies have already reported an increase incidence of triple negative breast cancers in obese women, statistical significance was only achieved in premenopausal: obese premenopausal women have 42% increased risk to develop triple negative tumours [37]. Our study observed that overweight and obese women have a decreased incidence of triple negative tumours which can be associated with the menopausal status, a limitation of our study, further discussed. Obesity is the cause of increase levels of estrogen in postmenopausal women that consequently increases the risk for ER + breast cancer by estrogen-driven mechanisms [35]. Several studies already observed a statistical association between ER + breast cancers and BMI categories [38–40]. Independent HER2 expression was found to be inversely associated to BMI in postmenopausal women [14] but increase levels of HER2 in combination with PR expression, were directly correlated with BMI in postmenopausal women and inversely correlated in premenopausal women [41]. Although we do not have data regarding menopausal status, our analysis did find an association with progesterone receptor. PR + was previously associated with obesity in postmenopausal population [42] although the role of PR in obese premenopausal women is yet to be uncover. Literature states that PR is expressed in approximately 75% of ER + breast tumours [43], accordantly, our data identified 1347 women with ER + /PR + and 197 women with ER + /PR−, which represents approximately 85% of ER + with PR +. Interestingly, a study performed in animal models observed that an increased expression of progesterone receptor in obese animals is associated with increased expression of tumour glycolytic and lipogenic enzymes, and proliferation markers [44]. These results were unexpected since obesity is mainly associated with estrogen activity through aromatase expression. Previous studies concluded that PR is a critical transcription regulator and an activator of several transduction pathways connected to the proliferative index [45]. Another suggested mechanism is the mitogenic potential of progestins (form of progesterone) that are able to enhance the proliferation rate [45]. We postulate that this association could be linked to an important feature in cancer in patients with obesity that is the high proliferative status.

Tumour site has been suggested to be a prognostic factor, tumours developed in the inferior quadrants tend to have a worst prognosis [46]. To the best of our knowledge, no studies have associated BMI and topographic localization, although ours is a negative result. Regarding laterality, there is a slight ratio tendency for left sided breast tumours to be more prevalent as it was found in our cohort. Left sided tumours seem to have a worse prognosis probably associated with cardiotoxicity, a side effect from radiotherapy to the left chest wall/breast [47].

Regarding outcomes, we found a low prevalence rate of 1.8% of BBC concordant with the literature (1.4 to 11.8%) [48], and found that overweight women are more likely to develop BBC than obese women. An association between obesity and BBC was already proposed, Zhang and colleagues compared 512 BBC patients with 1024 UBC and concluded that obesity has a significant impact on the survival outcomes of patients with BBC [49].

Association studies between BMI and differentiation grade are controversial, several studies found no significant relationship between histopathology grading and BMI [50, 51], but numerous other studies found obesity to be associated with poorly differentiation tumours [52, 53]. Our results reveal that obese women have statistically significant increase likelihood to develop poorly differentiated tumours with a OR of 48%, a study from Stark and colleagues found an even higher *OR* (~ 80%) for women with obesity to develop poorly differentiated tumours with a more advanced pathological stage [52].

Epidemiological evidence agrees that patients with obesity display a poorer prognosis with a more advanced stage disease at diagnosis, which includes larger tumours. Neuhouser et al. reported that obesity present a *OR* of 2.12 for larger tumours [12], and although with a lower OR, our results are coherent with the literature. Moreover, these findings can be connected to the fact that obese patients are diagnosed later than normoponderal breast cancer patients, hence presenting larger primary tumours. In our cohort, overweight and obese women are less prone to develop metastatic cancer, we hypothesize that it could be related to a metabolic paradox found in breast cancer patients associated with the diabetic state. High levels of circulating insulin can display protective menopausal-dependent mechanisms in breast cancer [54]. Unfortunately, we were unable to confirm such hypothesis due to the study limitations (further discussed), namely the absence of information regarding insulin levels and menopausal status.

Strong evidence from epidemiological studies also supports the fact that obesity is associated with lower overall and breast free disease survival in several transversal populations [23, 24], we also observed the same result, although without statistical significance.

Nevertheless, we are conscious of our study limitations. Some considerable information was not made available for different reasons. Biomarkers that were not routinely recorded like blood insulin, estrogen, or progesterone levels. Although we had access to the date from the last clinical follow-up, we consider that some of these data could be outdated due to the constrictions derived from the pandemic condition. We also couldn’t access information regarding the patient’s menopausal status and other comorbidities. We cannot rule out the possibility that these patients exhibit other metabolic comorbidities that are often clustered together with obesity, including hyperglycaemia, insulin resistance, dyslipidaemia, hypertension, which are known to result in increased mortality rates.

We observed that overweight condition should be considered as an important and independent entity. Despite in, most analysis, the overweight group is closer to the obese group, the former group behaves in a distinct manner, for instances in histological type and bilaterality. We realized that our results mostly corroborate the literature, that supports the significance modulation of obesity in breast cancer. Nonetheless, our findings further show that the three patient groups unravel distinct behaviours, which represent different prognostic features and therapeutical approaches that need to be addressed in the future.

## Supplementary Information

Below is the link to the electronic supplementary material.

Supplementary file1 (DOCX 16 KB)

Supplementary file2 (DOCX 45 KB)

Supplementary file3 (DOCX 14 KB)

Supplementary file4 (DOCX 14 KB)

## Data Availability

The datasets generated during and/or analysed during the current study are not publicly available due to privacy or ethical restrictions.
