# Supplementary material for: A retrospective study in tumour characteristics and clinical outcomes of overweight and obese women with breast cancer
Source: Breast Cancer Res Treat. 2022 Dec 28;198(1):89–101. doi: 10.1007/s10549-022-06836-5 (PMC9883351; doi:10.1007/s10549-022-06836-5)
Supplement: Supplementary file 1 — Supplementary file1 (DOCX 16 KB) [file 10549_2022_6836_MOESM1_ESM.docx]

Supplementary Information SI1 – Multinomial logistic regression for topographic localization

| Reference category: Multiple | | p-value | OR | 95% CI | |
| --- | --- | --- | --- | --- | --- |
|  |  |  |  | Lower | Upper |
| SIQ | Intercept | 0.004 | - | - | - |
|  | Normal | - | 1 | - | - |
|  | Overweight | 0.366 | 1.178 | 0.825 | 1.683 |
|  | Obese | 0.923 | 0.980 | 0.654 | 1.469 |
| SOQ | Intercept | 0.016 | - | - | - |
|  | Normal | - | 1 | - | - |
|  | Overweight | 0.728 | 0.960 | 0.760 | 1.211 |
|  | Obese | 0.325 | 0.879 | 0.679 | 1.137 |
| IIQ | Intercept | 0.003 |  |  |  |
|  | Normal | - | 1 | - | - |
|  | Overweight | 0.587 | 0.872 | 0.532 | 1.429 |
|  | Obese | 0.940 | 1.020 | 0.604 | 1.723 |
| IOQ | Intercept | 0.078 | - | - | - |
|  | Normal | - | 1 | - | - |
|  | Overweight | 0.092 | 0.679 | 0.434 | 1.065 |
|  | Obese | 0.641 | 0.895 | 0.560 | 1.429 |
| Other | Intercept | 0.000 | - | - | - |
|  | Normal | - | 1 | - | 0 |
|  | Overweight | 0.708 | 1.089 | 0.696 | 1.704 |
|  | Obese | 0.953 | 0.985 | 0.603 | 1.610 |

Adjusted to age at diagnosis and family history; Legend: IOQ – Inferior outer quadrant; IIQ – Inferior inner quadrant; SOQ – Superior outer quadrant; SIQ – Superior inner quadrant; CI – Confidence interval; OR – Odd ratio.
