# Supplementary material for: A retrospective study in tumour characteristics and clinical outcomes of overweight and obese women with breast cancer
Source: Breast Cancer Res Treat. 2022 Dec 28;198(1):89–101. doi: 10.1007/s10549-022-06836-5 (PMC9883351; doi:10.1007/s10549-022-06836-5)
Supplement: Supplementary file 2 — Supplementary file2 (DOCX 45 KB) [file 10549_2022_6836_MOESM2_ESM.docx]

Supplementary Information SI3 – Description of all histological types stratified by BMI categories

(NA – Not available)

References:

1. Nascimento RG do. Otoni KM. Histological and molecular classification of breast cancer: what do we know? *Mastology*. 2020;30. doi:10.29289/25945394202020200024

2. McCart Reed AE. Kalinowski L. Simpson PT. Lakhani SR. Invasive lobular carcinoma of the breast: the increasing importance of this special subtype. *Breast Cancer Research*. 2021;23(1). doi:10.1186/s13058-020-01384-6

3. Zhao H. The prognosis of invasive ductal carcinoma. lobular carcinoma and mixed ductal and lobular carcinoma according to molecular subtypes of the breast. *Breast Cancer*. 2021;28(1). doi:10.1007/s12282-020-01146-4

4. Makki J. Diversity of breast carcinoma: Histological subtypes and clinical relevance. *Clinical Medicine Insights: Pathology*. Published online 2015. doi:10.4137/CPath.s31563

5. Limaiem F. Mlika M. *Cancer. Tubular Breast Carcinoma*.; 2019.

6. Vranic S. Skenderi F. Beslagic V. Gatalica Z. Glycogen-rich Clear Cell Carcinoma of the Breast: A Comprehensive Review. *Applied Immunohistochemistry and Molecular Morphology*. 2020;28(9). doi:10.1097/PAI.0000000000000850

7. Trevisi E. la Salvia A. Daniele L. et al. Neuroendocrine breast carcinoma: a rare but challenging entity. *Medical Oncology*. 2020;37(8). doi:10.1007/s12032-020-01396-4

8. Hassan Z. Boulos F. Abbas J. el Charif MH. Assi H. Sbaity E. Intracystic papillary carcinoma: clinical presentation. patterns of practice. and oncological outcomes. *Breast Cancer Research and Treatment*. 2020;182(2). doi:10.1007/s10549-020-05680-9

9. Rissanen PM. Holsti P. Paget’s disease of the breast: The influence of the presence or absence of an underlying palpable tumor on the prognosis and on the choice of treatment. *Oncology (Switzerland)*. 1969;23(3). doi:10.1159/000224484

10. Nagarajan B. Autkar G. Patel K. Sanghvi M. Primary breast liposarcoma. *Journal of Radiology Case Reports*. 2018;12(12). doi:10.3941/jrcr.v12i12.3457

11. Ai D. Sweeney K. Li X. Short review of malignant adenomyoepithelioma of the breast. *Human Pathology Reports*. 2022;27. doi:10.1016/j.hpr.2021.300583

12. Jagsi R. Mason G. Overmoyer BA. et al. Inflammatory breast cancer defined: proposed common diagnostic criteria to guide treatment and research. *Breast Cancer Research and Treatment*. 2022;192(2). doi:10.1007/s10549-021-06434-x

13. Jadhav T. Prasad SS. Guleria B. Tevatia MS. Guleria P. Solid papillary carcinoma of the breast. *Autopsy and Case Reports*. 2022;12. doi:10.4322/acr.2021.352
