# Supplementary material for: A retrospective study in tumour characteristics and clinical outcomes of overweight and obese women with breast cancer
Source: Breast Cancer Res Treat. 2022 Dec 28;198(1):89–101. doi: 10.1007/s10549-022-06836-5 (PMC9883351; doi:10.1007/s10549-022-06836-5)
Supplement: Supplementary file 3 — Supplementary file3 (DOCX 14 KB) [file 10549_2022_6836_MOESM3_ESM.docx]

Supplementary Information SI4 – Cox proportional hazard models for overall survival

|  | Crude | | | | Adjusted^§^ | | | |
| --- | --- | --- | --- | --- | --- | --- | --- | --- |
|  | p-value | HR | 95% CI | | p-value | HR | 95% CI | |
|  |  |  | Lower | Upper |  |  | Lower | Upper |
| BMI | 0.186 | - | - | - | 0.245 | - | - | - |
| Normal | - | 1 | - | - | - | 1 | - | - |
| Overweight | 0.174 | 1.224 | 0.915 | 1.637 | 0.183 | 1.224 | 0.909 | 1.648 |
| Obese | 0.079 | 1.336 | 0.967 | 1.847 | 0.117 | 1.309 | 0.934 | 1.833 |

^§^Adjusted to Age at diagnosis, Family history, laterality, Topographic localization, histological type, and Receptor status. Legend: HR – Hazard ratio; CI – Confidence interval
